# Immunoglobulin G4-related diseases in the head and neck: a systematic review

**DOI:** 10.1186/s40463-015-0071-9

**Published:** 2015-06-20

**Authors:** Graeme B. Mulholland, Caroline C. Jeffery, Paras Satija, David W. J. Côté

**Affiliations:** Division of Otolaryngology-Head and Neck Surgery, 1E4 Walter MacKenzie Centre, University of Alberta, 8440 112 Street, Edmonton, AB T6G 2B7 Canada

**Keywords:** IgG4-RD, Head and neck, Systematic review, Salivary glands, Lacrimal glands, Lymphandenopathy

## Abstract

**Background:**

Immunoglobulin G4 related disease (IgG4-RD) is a poorly understood chronic inflammatory disorder affecting the middle-aged and elderly that can present to the otolaryngologist. We aim to summarize the current literature regarding the manifestations and management of IgG4-RD in the head and neck.

**Methods:**

Pubmed and EMBASE were searched using the term relevant search algorithm utilizing keywords such as: IgG4 related disease, head and neck, orbit, salivary glands, sialadenitis, Kuttner, angiocentric eosinophilic fibrosis, submandibular, lacrimal, thyroid, dacryoadenitis, nasal, sinus, and Mikulicz’s. Reference lists were searched for identification of relevant studies.

Case reports, original research and review articles published in English from 1964 to 2014 whose major topic was IgG4-RD affecting the head and neck were included. Data regarding patient demographics, presentation, histopathology, management and treatment outcomes of IgG4-RD were extracted. Level of evidence was also assessed and data were pooled where possible. Three independent reviewers screened eligible studies; extracted relevant data and discrepancies were resolved by consensus, where applicable. Descriptive and comparative statistics were performed.

**Results:**

Fourty-three articles met our inclusion criteria. IgG4-RD most often presents as a mass lesion in the head and neck region. Common diagnostic features include: 1) elevated serum IgG4 level, 2) marked infiltration of exocrine glands by IgG4-positive plasma cells with fibrosis, and 3) marked improvement with corticosteroid therapy and additional immunosuppressive therapy in corticosteroid refractory cases. Early diagnosis and involvement of rheumatology is important in management.

**Conclusions:**

IgG4-RD is a challenging non-surgical disease that has multiple manifestations in the head and neck. It must be distinguished from various mimics including malignancy, systemic diseases, and infectious. Otolaryngology-Head and Neck surgeons should be aware of this condition and its management.

## Introduction

Immunoglobulin G4 – related disease (IgG4-RD) is a newly described fibroinflammatory condition that often presents as a tumefactive lesion that can affect nearly every organ system. IgG4-RD was first recognized after a connection between elevated serum IgG4 levels and inflammatory mass lesions in the pancreas causing autoimmune pancreatitis was made by Hamano *et al.* in 2001 [1]. An initial consensus statement regarding diagnosis of IgG4-RD was developed by Deshpande *et al.* at the first international symposium for IgG4-RD held in October of 2011 [2]. After the pancreas, the head and neck region is second most common site for presentation of IgG4-RD. More, a number of historically perplexing pseudotumor disorders have been attributed to IgG4-RD; these include Mikulicz’s disease, Küttner’s tumor and Reidel’s thyroiditis [3].

The exact etiology of IgG4-RD is unknown and no known role of the IgG4 molecule itself has been identified. It is postulated that the inflammatory and fibrotic processes that drives IgG4-RD are propagated by a combination of Th2 cells and regulatory T cells (Treg cells) [4]. This is contrary to most autoimmune disorders where polarized T helper 1 (Th1) and/or Th17 subsets are responsible for the inflammatory process [5]. Histologically, the hallmark findings for IgG4-RD include lymphoplasmacytic infiltration, storiform fibrosis, obliterative phlebitis, and mild to moderate tissue eosinophilia [6]. However, the exact histological findings vary greatly depending on the tissue affected and clinical presentation. Currently, the histologic diagnosis of IgG4-RD is based primarily on IgG4 positive to IgG containing cell ratio and the number of IgG4 positive cells per high powered field, a ratio of IgG4 to IgG that is higher than 50 % and 30 IgG4-positive cells per high-power field is considered to be highly suggestive of IgG4-RD [6].

Currently, the literature proposes that IgG4-RD could be both over and under recognised [7]. This study aims to examine the various presentations of IgG4-RD in the head and neck, and present the management and outcomes reported in the literature.

## Material and methods

This systematic review was performed using the following search strategy and study selection criteria.

### Literature search strategy

The databases PubMed (1966-December 2014) and Embase (1988-December 2014) were searched using an algorithm designed from an extensive list of relevant search terms (see Appendix A for Pubmed and Embase search algorithms).

We included all original studies, case reports, case series, and reviews. Relevant articles and abstracts were selected and reviewed and the reference lists from these sources and recent review articles were also reviewed for additional publications.

### Study selection

Three independent reviewers screened the identified articles (GBM, CCJ and PS). Relevant articles were obtained and reviewed in full. Discrepancies were resolved by consensus amongst the reviewers. The inclusion criteria comprised of all original clinical studies, case series and case reports of histologically confirmed IgG4-RD in the head and neck. Histologic diagnosis of IgG4-RD required identification of >10 IgG4 positive plasma cells per high powered field, IgG4 + plasma cell to IgG containing cell ratio 40 % or greater, and characteristic finding of fibrosis, sclerosis and phlebitis. Articles were excluded on the basis of biopsy information not being from a head and neck site, insufficient histologic information, inability to extract head and neck specific information and narrative review and expert opinions.

### Data extraction

The information gathered for each study included study design, country of publication and number of patients. Where possible, patient-specific data was extracted, including age at presentation, duration and nature of symptoms. Specifically, we collected data regarding head and neck manifestations, laboratory and histologic findings as well as specific treatments and outcome information.

### Statistical analysis

Basic statistical analysis, including descriptive statistics was performed using Excel (Version 19.0, Microsoft ®).

## Results

**Fig. 1:**
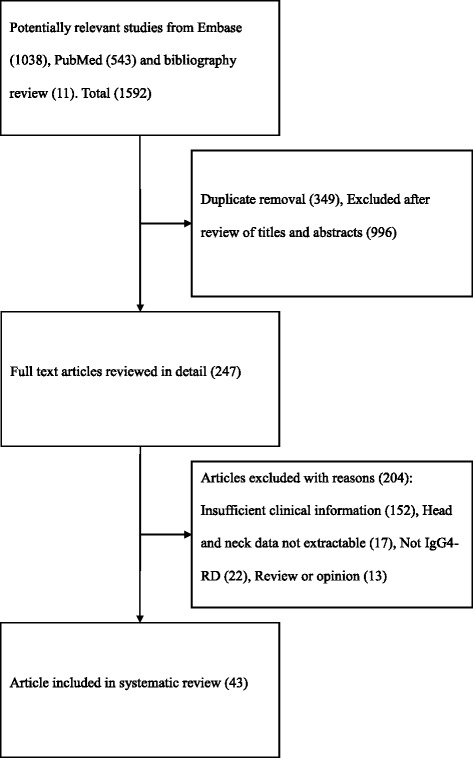
Flow chart of studies obtained through literature search, eligible and excluded (Uploaded separately as per submission instructions)

We initially identified 1592 articles through a combination of literature search and citation review. After reviewing abstracts, 247 articles of interest were identified. Three authors independently reviewed the articles and 43 articles met inclusion criteria (Fig. 1). Of the 43 articles, 21 case reports and 5 large case series (greater than 15 patients) were included (Table 1). A large proportion of articles were from Japan (41.9 %), contributing 86.6 % of the total individual cases (Table 1).

**Table 1:** Country of origin and characteristics of included articles

			N (Articles)	%	N (Individual Cases)	%
Total number of studies			43		484	
	Case Reports		21	48.8	21	4.3
	Small Case Series		17	39.5	107	22.1
	Large Case Series		5	11.6	356	73.6
Country of Origin	North America		10	23.3	27	5.6
		Canada	1	2.3	1	0.2
		USA	9	20.9	26	5.4
	Asia		22	51.2	429	88.6
		Japan	18	41.9	419	86.6
		Hong Kong	2	4.7	8	1.7
		Taiwan	1	2.3	1	0.2
		Singapore	1	2.3	1	0.2
	Oceania		6	14.0	7	1.4
		Australia	5	11.6	6	1.2
		New Zealand	1	2.3	1	0.2
	Europe		4	9.3	20	4.1
		United Kingdom	1	2.3	1	0.2
		Netherlands	1	2.3	12	2.5
		Czech Republic	1	2.3	6	1.2
		Switzerland	1	2.3	1	0.2
	South America		1	2.3	1	0.2
		Brazil	1	2.3	1	0.2

Four hundred and eighty-four patients were identified with an average patient age of 60.4 years (Table 2). Table 3 shows the proportion of patients presenting by various sites in the head and neck. Cervical lymphadenopathy was document in 22 cases. Many of the patients also had involvement of other organ systems at the time of presentation, including 56 patients with lymphadenopathy outside of the head and neck (see Table 3). All patients had tissue biopsy from head and neck sites confirming the diagnosis of IgG4-RD. Confirmatory laboratory investigations was included where documented. The laboratory and histologic findings of patients indentified in the literature are summarized in Table 4.

**Table 2:** Basic patient demographics

Total number of Patients	484
Average Age (years)	60.4
Percent Males	47.5 %

**Table 3:** Systemic and head and neck manifestations

Head & Neck Manifestations	Site	Subsite	n	%
	Total presentations		730	
	Orbit		384	52.6
		Lacrimal Gland	136	18.6
		Extra Ocular Muscles	9	1.2
		Optic Nerve	5	0.7
	Salivary Glands		162	22.2
		Submandibular Gland	107	14.7
		Parotid Gland	29	4.0
		Sublingual Gland	1	0.1
		Minor Salivary Gland	1	0.1
	Thyroid		31	4.2
	Facial Skin		6	0.8
	Trigeminal Nerve		1	0.1
	Cervical Lymphadenopathy		22	3.0
Non Head & Neck Manifestations	Site		n	%
	Other Organ Involvement		68	9.3
	Mediastinal/Pelvic Lymphadenopathy		56	7.7

**Table 4:** Laboratory and histologic findings

	Mean Values
Serum IgG4 (mg/dL)	702.9
Serum IgG (mg/dL)	2445.0
IgG4 + ve Plasma Cells / HPF	69.8
IgG4 + ve Cells / IgG Containing Cells	48.0 %

Treatment information was available for 26.7 % of patients. Patients were treated with surgical excision, radiotherapy, corticosteroids with or without adjunct medical treatment, or some combination of treatment modalities. The distribution of patients in terms of treatment(s) received and their outcomes are summarized in Table 5. Treatment outcomes were known in 99 patients. Of these, full remission was seen in 90.0 % in response to medical treatment. Corticosteroids treatment alone was effective for 67 patients (67.7 %) in achieving full remission.

**Table 5:** Treatment and progress

Treatments Received by Patients (*N* = 129)		n	%
Surgical Management Alone		15	11.6
Combined Surgical and Medical Management		4	3.1
Medical Management Alone		107	82.9
	Treatment with Corticosteroids^a^ Alone	84	65.1
	Treatment with Corticosteroids and One Additional Immunosuppressive Agent^b^	16	12.4
	Treatment with Corticosteroids and Multiple Additional Immunosuppressive Agents	10	7.8
Treatment Outcomes (*N* = 99)		n	%
Full Remission with Medical Treatment		89	90.0
Full Remission with Corticosteroids Alone		67	67.7
	Intolerance, Relapse with Taper, or Treatment Failure with Corticosteroids as First Line	32	32.3
Full Remission with Addition of Single Immunosuppressive Agent to Corticosteroids		14	14.1
Full Remission with Addition of Multiple Immunosuppressive Agents to Corticosteroids		8	8.1
Remission Not Achieved		9	9.1

^a^Corticosteroids included: prednisolone, methylprednisone and triamcinolone injections

^b^Additional immunosuppressive agents included: rituximab, methotrexate, azathioprine, mycophenolate mofetil, tamoxifen, 6-mercaptopurine, chlorambucil, cyclosporine and cyclopohosphamide

## Discussion

This is the first systematic review of IgG4-RD presentation in the head and neck. Our study demonstrates a strong propensity for IgG4-RD to present in the head and neck region. We included 43 articles containing 484 patients. The most common site of presentation was the orbit, followed by the submandibular gland – with many patients having presentations in multiple head and neck and distant sites. Treatment information was also collected, showing that majority of patients receiving corticosteroids responded very well to treatment.

The vast majority of the included cases were from Asia (429 of 484 or 88.6 %) and more specifically Japan (419 of 484 or 86.6 %). Indeed, much of the literature on IgG4-RD originates from Japan [8]. This poses the question of whether IgG4-RD more prevalent in the Japanese population or simply better recognized? While IgG4-RD was first recognized in Japan, it is increasingly recognized throughout the rest of the world [7]. While cases have been reported on every continent and in most ethnic groups, reports from countries outside of Asia comprise smaller case series. This highlights the emerging status of IgG4-RD in the literature and likely an increase in reporting in the future.

While systemic presentations of IgG4-RD favor males over females with a reported ratio of 2.8-3.5:1, our study demonstrates an almost 1 to 1 ratio of head and neck manifestations [6, 9, 10]. Orbital involvement was most common subsite in the head and neck and the majority of the orbital presentations (219 cases) came from a single study [11]. Common orbital manifestations included periorbital swelling, eyelid swelling, and proptosis. Salivary gland and lacrimal gland involvement were very common and included submandibular, parotid gland, and lacrimal gland enlargement, infiltration, and formation of pseudotumours. Lymphadenopathy was a particularly common presentation in the head and neck. This was often associated with lymphadenopathy elsewhere including the mediastinum and retroperitoneum. More rare forms of head and neck involvement included the thyroid gland in the form of Riedel’s Thyroiditis as well as sinonasal and airway manifestations.

Few patients in our series received surgical excision alone. The majority cases received some form of medical management comprising of high-dose corticosteroids. Patients had excellent response to medical therapy alone with full remission rate of 90 %. A consensus statement from 17 referral centres in Japan developed a treatment regime of 0.6 mg/Kg prednisolone for 2 to 4 weeks with a taper over 3 to 6 months and a low daily dose for 3 years. Importantly, this regime was developed for treatment of autoimmune pancreatitis, where consequences of not treating are associated with significant morbidity and mortality [12]. However, other authors advocate watchful waiting with observation over a number of years as an acceptable treatment approach [13]. Based on our results, surgery remains most useful for obtaining histologic diagnosis.

Strict selection criteria was used in article selection. Histologic diagnosis was based on IgG4 + ve cells/ HPF and the IgG4 + ve/IgG ratio. This is considered the most rigorous definition of IgG4-RD [4, 6]. However, since biopsy from the head and neck was one of the criteria, there are likely many studies of IgG4-RD in the head and neck that were excluded as biopsies were obtained from other tissues. Unfortunately, there is a paucity of high quality publications on this topic. The majority of the information available exists in the form of case reports and small case series, which comprise of low level of evidence. There are also inconsistencies in reporting key information. Many studies were excluded due to insufficient information (152 or 74.5 %) or a lack of basic histologic information; articles lacking IgG4+ cell/HPF or the IgG4+/IgG ratio --items critical to confirming the presence of IgG4-RD-- were excluded.

## Conclusions

Due to the numerous potential manifestations of IgG4-RD in the head and neck, it is crucial for otolaryngologists to be aware of this condition. A high index of suspicious is required particularly in the setting of patients who present with recurrent salivary and lacrimal gland swelling, lymphadenopathy, along with fibroinflammatory systemic involvement. This disease process remains under recognized and poorly understood. Future studies are necessary to better understand the pathophysiology and natural history of this disease.

## Appendix A: search strategy

Ovid: Embase search:

1. exp salivary gland/
2. mouth disease/ or lip disease/ or exp mouth tumor/ or exp palate disease/ or exp pharynx disease/ or exp salivary gland disease/ or exp tongue disease/ or xerostomia/
3. head/ or exp “face, nose and sinuses”/ or exp skull/
4. “head and neck disease”/ or exp “head and neck tumor”/
5. neck/
6. exp lacrimal apparatus/
7. exp lacrimal gland disease/
8. exp ear nose throat disease/
9. angiocentric eosinophilic fibrosis.mp.
10.  (Facial or eyelid* or mouth or oral or gingival or lip or lips or palate or palatal or tonsil* or sinuses or sinus cavit* or salivary gland* or tongue or otorhinolaryngologic or ear or ears or larynx or laryngeal or nose or noses or paranasal or pharyngeal or parathyroid or thyroid or tracheal or sialadenitis or kuttner or submandibular or lacrimal or thyroid or dacryoadenitis or orbit or mikulicz*).ti,ab.
11.  or/1-10
12.  (immunoglobulin g4 or igg4).mp.
13.  11 and 12
14.  limit 13 to english language
15.  limit 14 to exclude medline journals
16.  14 not 15
17.  limit 16 to (conference abstract or conference paper or conference proceeding or “conference review”)
18.  14 not 17
19.  limit 18 to embase

Ovid: Pubmed search:

1. exp Salivary Gland Diseases/
2. exp Head/
3. Neck/
4. exp Lacrimal Apparatus Diseases/ or exp Lacrimal Apparatus/ or exp Lacrimal Duct Obstruction/
5. angiocentric eosinophilic fibrosis.mp.
6. nose diseases/ or granuloma, lethal midline/ or nasal obstruction/ or exp nose neoplasms/ or exp paranasal sinus diseases/
7. orbital diseases/ or exp exophthalmos/ or orbital pseudotumor/
8. exp Frontal Sinus/ or exp Sphenoid Sinus/ or exp Maxillary Sinus/ or exp Pyriform Sinus/ or exp Ethmoid Sinus/
9. (Facial or eyelid* or mouth or oral or gingival or lip or lips or palate or palatal or tonsil* or sinuses or sinus cavit* or salivary gland* or tongue or otorhinolaryngologic or ear or ears or larynx or laryngeal or nose or noses or paranasal or pharyngeal or parathyroid or thyroid or tracheal or sialadenitis or kuttner or submandibular or lacrimal or thyroid or dacryoadenitis or orbit* or mikulicz*).mp.
10.  (immunoglobulin g4 or igg4).mp.
11.  or/1-9
12.  10 and 11
13.  limit 12 to english language
14.  remove duplicates from 13
